# Multiomics analysis revealed the temporally common and specific molecular changes in *Arabidopsis thaliana* (L.) under salt stress

**DOI:** 10.1186/s12864-025-12292-4

**Published:** 2025-11-19

**Authors:** Zixuan Chen, Chanjuan Ye, Yuan Zeng, Jie Guo, Xinqiao Zhou, Dagang Chen, Juan Liu, Chuanguang Liu, Mariusz Jaremko, Ke Chen, Guoqiang Fan

**Affiliations:** 1 College of Forestry, Henan Agricultural University, Zhengzhou, Henan 450002 China; 2 Rice Research Institute, Guangdong Academy of Agricultural Sciences; Guangdong Key Laboratory of Rice Science and Technology; Guangdong Rice Engineering Laboratory; Key Laboratory of Genetics and Breeding of High Quality Rice in Southern China (Co-construction by Ministry and Province), Ministry of Agriculture and Rural Affairs, Guangzhou, Guangdong 510640 China; 3 Southern Piedmont Agricultural Research and Extension Center, Virginia Tech, Blackstone, VA 23824 United States; 4 The Golden Ratio Institute, Riyadh, 13244 Kingdom of Saudi Arabia

**Keywords:** *Arabidopsis thaliana*, Salt stress, Multiomics analyses, Dynamic changes

## Abstract

Salt stress is a major abiotic constraint that limits plant growth and productivity worldwide. In this study, we performed a comprehensive temporal analysis using transcriptomics (6 h), ribosome profiling (12 h), proteomics and phytohormone quantification (24 h), and metabolomics (48 h) to uncover the regulatory mechanisms in *Arabidopsis thaliana* underlying salt stress adaptation. Novel transcriptional regulators, including *JAZ7*, *CBF4*, *bHLH92*, and *NAC041* that responded rapidly to early salt stress, were identified. At the post-transcriptional level, *TAS1C* and *TAS2*, along with chloroplast tRNAs (*AtTRNR.1*, *AtTRNC*, *AtTRNV.1*), were found to be translationally upregulated, suggesting a previously unrecognized role of organellar translation in stress response. At the protein level, chloroplast functional proteins, AtPSBA, AtRBCL, AtPSAA, AtPSAB, were revealed to respond to salt stress. Some functional proteins, including AtCER1, AtGGL19, and AtLEA14, with opposite trends between transcription and translation, highlighting the complexity of salt stress adaptation. Abscisic acid (ABA) was significantly upregulated, while jasmonic acid (JA) was dramatically suppressed, with AtOPR3 and JAZ7 identified as key regulatory nodes. Metabolomics analysis further showed that d-proline and 1-pyrroline-2-carboxylate accumulated at later stages, potentially contributing to increased salt stress resistance. Overall, these findings provide new insights into the temporal regulation of stress adaptation and identify candidate genes and metabolites that may serve as targets for improving salt tolerance in crops.

**Supplementary Information:**

The online version contains supplementary material available at 10.1186/s12864-025-12292-4.

## Highlights

A multiomics analysis revealed both common and specific regulatory responses to salt in *A. thaliana*. The results of this study can improve our understanding of the interconnected signaling networks in plants, linking transcriptional, post-transcriptional, proteomics, phytohormonal, and metabolic pathways.

**Supplementary Information:**

The online version contains supplementary material available at 10.1186/s12864-025-12292-4.

## Introduction

As sessile species, plants continuously adapt to a wide range of abiotic and biotic stresses, such as soil salinity, drought, temperature extremes, nutrient deficiency, toxic metal accumulation, radiation, microbial infections, and herbivore attacks [1, 2]. Over the course of evolution, plants have developed diverse strategies to cope with these environmental challenges and defend against pathogen and pest invasions. Among abiotic stresses, salt stress is particularly detrimental, affecting approximately 45 million hectares of irrigated land worldwide [3, 4]. In recent years, extensive research on *A. thaliana* has advanced our understanding of the molecular basis of salinity tolerance [5]. Nevertheless, the biological functions of several salt-responsive genes remain poorly characterized, and the regulatory networks connecting them are not yet completely understood. For instance, abscisic acid (ABA) rapidly accumulates under salt stress, triggering stomatal closure and Na^+^ sequestration [6]. The *AtC3H3* has been reported to regulate plant salt stress by influencing the expression of salt stress-responsive genes, such as *Responsive to Desiccation 29B* (*RD29B*), *Dehydration-Responsive Element Binding protein 2A* (*DREB2A*) and *DREB2B* [7]. Another gene, *AtDPBF3*, encoding a key member of the ABI5 subfamily, positively regulate salt stress response through alterations of gene expression in *AtCXX5*, *AtSOS3*, *AtSUT4*, and *AtLEA4-5* etc. [8].

In addition to ABA, Jasmonic acid (JA) is similarly crucial [9]. In *A. thaliana*, lipoxygenase 3 (LOX3) expression was dramatically induced by salt treatment [10]. In maize, the APETALA2/ethylene-responsive factor (AP2/ERF)-domain transcription factor ZmEREB57, activates *ZmAOC2* to accelerate 12-oxo-phytodienoic acid (OPDA)/JA synthesis and improve maize salt tolerance [11], and high salinity promotes JASMONATE ZIM domain 8 (JAZ8) degradation, releasing the heterotrimeric NF–YA1–YB2–YC9 complex that activates *MYB75* [12]. Together, these molecular modules reprogram transcription and re-establish hormone homeostasis, forming the backbone of plant salt adaptation.

Omics-based approaches have emerged as essential technologies for developing crops with improved quality [13, 14] and for revealing the dynamic regulation of biological processes across developmental stages and environmental conditions [15, 16]. These approaches also hold considerable promise for identifying novel *cis*-regulatory elements and stress-responsive regulatory modules. Recent studies integrating multi-omics has identified novel transcription factors improving salinity tolerance in rice [17], revealed the roles of RVE8-like proteins in major cellular processes [18], and provided insights into species-specific metabolic traits in chia (*Salvia hispanica*) [19]. Although these omics approaches, alone or in combination, have become mainstream in *A. thaliana* research, studies that integrate temporal transcriptomics, ribo-seq, proteomics, phytohormone profiling, and metabolomics to resolve multilevel salt response remain limited.

In this study, we applied a multiomics approach, including transcriptomics, ribosome profiling, proteomics, metabolomics, and phytohormone analyses, to characterize the dynamic and temporal responses of *A. thaliana* to salt stress. In the early stages of abiotic stress, transcription factors such as JAZ7 and CBF4 were rapidly upregulated, initiating signal transduction pathways in plants. At the post-transcriptional level, changes were detected in the expression of nuclear RNAs and photosynthesis-related genes, suggesting their roles in response to salt stress. Proteomics analysis revealed alterations in the abundance of functional enzymes, indicating a shift from normal growth processes to stress defense mechanisms in plants. Over a long time, only the levels of ABA were upregulated under salt stress, whereas those of other major phytohormones were downregulated. In the later stage of salt stress, the levels of secondary metabolites, such as d-proline and 1-pyrroline-2-carboxylate, were significantly altered, potentially contributing to long-term stress adaptation. By integrating RNA-seq, ribo-seq, and proteomics data across the salt-stress time series, we provide the first dynamic map of the transcription-translation-protein regulatory network in *A.thaliana.* Our study offers a temporal-specific resource for precision mining of salt-tolerance gene modules and a direct guidance for salt-resilient crop breeding.

## Methods and materials

### Plant material and growth conditions

**Fig. 1:**
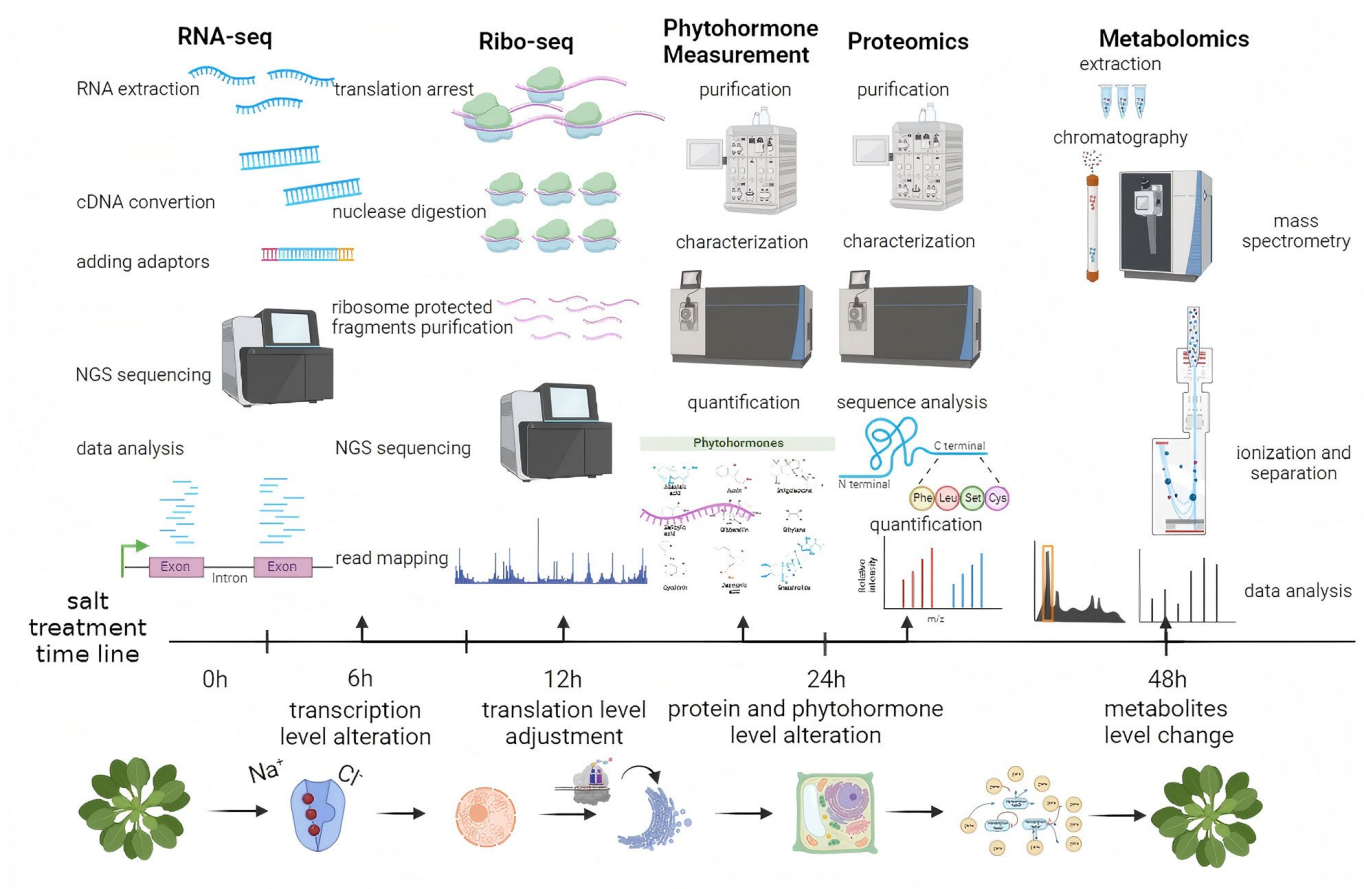
Overview of sample preparation and experimental workflow. *A. thaliana* seedlings were germinated and grown on 1/2 MS medium for two weeks, then transferred to either mock medium (1/2 MS) or salt medium (1/2 MS supplemented with 125 mM NaCl). Samples collected at 6 h post-treatment were used for RNA-seq analysis to assess transcriptome changes. Ribo-seq was performed on samples harvested at 12 h to evaluate translational dynamics. Proteomic and phytohormone analyses were conducted using samples collected at 24 h, while metabolomic profiling was carried out on samples collected at 48 h post-treatment

*A. thaliana* (L.) Columbia-0 (Col-0) seeds were obtained from the Arabidopsis Biological Resource Center. Seeds were surface-sterilized and germinated on 1/2 Murashige & Skoog (MS) medium [20]. After 2 weeks of growth, 30 uniformly developed seedlings were transferred into freshly prepared 1/2 MS medium (i.e., mock-treated control) and 1/2 MS medium supplemented with 125 mM NaCl (i.e., salt-treated). Treatments were applied for four time durations, i.e., 6, 12, 24, and 48 h (Fig. 1), under controlled growth chamber conditions with a 16 h light and 8 h dark photoperiod, 160 µmol photons m^−2^ s^−1^ light intensity, and a constant temperature of 24 °C. For each time point and treatment, three seedlings were harvested and pooled together as a biological replicate, immediately frozen in liquid nitrogen, and stored at − 80 °C for subsequent experiments. Three independent biological replicates were collected for each treatment and time point.

### RNA-seq

Based on signaling transduction timelines and stress response dynamics, we anticipated differences in transcript profiles between salt-treated and mock-treated *A. thaliana* to occur at 6 h post treatment [21]. Therefore, 6 h after treatment, 2 g of whole plants was harvested per biological replicate for extracting total RNA using TRIzol Reagent (Invitrogen®, Thermo Fisher Scientific, California, U.S.) according to the manufacturer’s instructions. Then, mRNA was purified using poly-T oligo-attached magnetic beads (NEB, S1419S, U.S.). Sequencing libraries were generated using NEBNext® Ultra™ RNA Library Prep Kit for Illumina® (NEB, U.S.) according to the manufacturer’s protocols. Briefly, the first-strand cDNA was generated from 1 µg mRNA using the NEBNext First Strand Synthesis kit (NEB, U.S.) using a random hexamer primer and M-MuLV Reverse Transcriptase (RNase H^−^). The second-strand cDNA was then synthesized using DNA polymerase I and RNase H. After adenylation of the 3ʹ ends of DNA fragments, NEBNext adaptors with hairpin loop structure were ligated. The adaptor-ligated cDNA products with approximately 250–300 bp in length were selected and purified using the AMPure XP system (Beckman Coulter, Beverly, U.S.). The quality of library was evaluated on the Agilent Bioanalyzer 2100 system, and the library was sequenced on an Illumina NovaSeq platform to generate 150-bp paired reads.

### Ribo-seq

Ribosome profiling was conducted to examine translational regulation in response to salt stress at 12 h post treatment, a time point previously reported to be associated with translational-level signaling events under salt stress [22, 23]. After 12-h salt treatment, *A. thaliana* seedlings were used for ribosome profiling as described previously [24, 25]. Briefly, frozen samples were homogenized in 400 μL of lysis buffer (1% Triton X-100, chloramphenicol (34 mg mL^−1^), cycloheximide (50 mg mL^−1^), and DNase I (2 units mL^−1^). The lysates were then treated with RNase I, and ribosome-protected fragments (RPFs) were isolated using size-exclusion chromatography with MicroSpin S-400 HR columns. After rRNA depletion and PAGE purification, gradient preparation and nuclease digestion were performed using fragmentation buffer, followed by proteolytic digestion of ribosome proteins with proteinase K. Subsequent sucrose gradient centrifugation and precipitation with release buffer were performed to isolate RPFs. Library construction was performed using the ARTseq™ Ribosome Profiling Kit (RPHMR12126). The resulting libraries were quantified using a Qubit®2.0 Fluorometer and adjusted to 1 ng µL^−1^. The insert size was confirmed using an Agilent 2100 Bioanalyzer, and the library concentration was further validated by qPCR. Libraries that fulfilled the quality criteria were sequenced on the Illumina NextSeq 2000 platform for paired-end sequencing at 150 bp read length to generate 15 Gb of raw data.

### Proteomics

After 24 h post treatment, total proteins were extracted from harvested *A. thaliana* seedlings from each experiment using the TCA/acetone precipitation method [26, 27]. Protein concentrations were determined using the bicinchoninic acid protein assay. To reduce disulfide bonds, 1 M DTT was added to each sample for incubation at 56 °C for 1 h to reach a final protein concentration of 100 mM before trypsin digestion. The trypsin-digested peptide mixture was labeled using tandem mass tag (TMT) proreagents [28], and the labeled samples were combined, desalted using the MonoSpin C18 column (GL Science, Japan), and dried under vacuum. The dried peptide mixture was redissolved in buffer A (10 mM ammonium formate dissolved in ddH_2_O, pH 10.0) and fractionated using a 1290 UPLC system (Agilent Corporation, Santa Clara, U.S.) equipped with a reverse-phase column under high pH conditions. Five fractions were collected and dried in a vacuum concentrator for low-pH nano-HPLC–MS/MS analysis.

For LC–MS/MS analysis, the fractions were resuspended in 40 μL buffer C (0.1% formic acid) and separated on an EASY-nLC 1200 system coupled to a Q-Exactive-HF-X mass spectrometer operated in data-dependent acquisition mode (full MS 350–18,00 m/z, 60,000 resolution; HCD-MS2 NCE 35%, dynamic exclusion 30 s).

### Phytohormone quantification

*A. thaliana* leaf samples harvested at 24 h after salt or mock treatment were subjected to phytohormone analysis. Standard compounds, including indole-3-acetic acid (IAA), ABA, gibberellic acid A_3_ (GA_3_), zeatin, salicylic acid (SA), and JA, were purchased from Sigma and Shanghai Bio-Technology Co., Ltd. The liquid nitrogen–ground leaf powders were dissolved in the mobile phase solution and analyzed using an Agilent 1290 HPLC coupled with a tandem AB Qtrap 6500 mass spectrometer system. Compounds were separated based on their retention times on the chromatography column and ionized via electrospray ionization (ESI). The ionized molecules were then analyzed using triple quadrupole mass spectrometry (Q1, Q2, and Q3). Each hormone was detected based on its unique mass-to-charge ratio, and quantitative data were obtained accordingly.

### Metabolomics

A previous study suggested that dynamic changes in plant metabolites occur at 48 h after salt treatment [29]. Therefore, we conducted metabolomics analysis at this time point. In total, 50 mg of tissue from mock-treated and salt-treated *A. thaliana* seedlings was harvested, flash-frozen in liquid nitrogen, homogenized, and sonicated with 1000 μL of extraction buffer (methanol:H_2_O, 3:1) with an isotopically labeled internal standard mixture. The homogenization and sonication cycles were repeated three times. The lysates were incubated for 1 h at − 40 °C, followed by centrifugation at 12,000 rpm for 15 min at 4 °C. The resulting supernatants were transferred to a fresh glass vial for LC–MS/MS analysis. A quality control (QC) sample was prepared by mixing an equal aliquot of supernatants from all samples. Separation and detection of metabolites were performed on a UHPLC system (Vanquish, Thermo Fisher Scientific, USA) with a UPLC HSS T3 column (2.1 mm × 100 mm, 1.8 μm) coupled to a Q-Exactive HF-X mass spectrometer (Orbitrap MS, Thermo Fisher Scientific, USA). The mobile phase consisted of 5 mM ammonium acetate and 5 mM acetic acid in water and acetonitrile. The autosampler was maintained at 4 °C, and the injection volume was set to 3 μl. The QE HF-X mass spectrometer was used to acquire MS/MS spectra on the information-dependent acquisition mode in the control of the acquisition software (Xcalibur, Thermo) to continuously evaluate the full-scan MS spectra. ESI was used in both positive and negative modes, with the following source parameters: sheath gas flow rate, 30 Arb; auxiliary gas flow rate, 10 Arb; capillary temperature, 350 °C; full MS resolution, 60,000; MS/MS resolution, 7500; collision energy, 10/30/60 in NCE mode; and spray voltage, 4.0 kV (positive) or − 3.8 kV (negative).

### Data processing, annotation, and statistical analyses

#### RNA-seq and ribo-seq analyses

High-quality reads were obtained by removing sequences containing adapters or ambiguous nucleotides, and low-quality reads (*i.e.*, the proportion of nucleotides with Q ≤ 20 is > 50% of an entire read) were obtained from raw reads using Trimmomatic [30]. Hisat2 v2.0.5 was used to map high-quality clean reads to the reference genome and the annotation file (ftp://ftp.arabidopsis.org/home/tair/Genes/TAIR10_genome_release/) [31]. For identifying the novel transcripts, all assemblies were compared with the reference annotation using StringTie2 [32]. The number of reads mapped to each gene was counted using FeatureCounts v1.5.0-p3 [33] to calculate the fragments per kilobase per million mapped reads value of each gene. Principal component analysis (PCA) was conducted on all samples to determine the overall transcript differences between treatments and the degree of variability between samples within the same treatment. Differentially expressed genes (DEG) between mock and salt treatments were identified using the DESeq2 package in R, and any genes with an adjusted *P* value of ≤ 0.05 assessed using the Benjamini–Hochberg (BH) method were considered DEGs [34]. A heatmap of the top 50 DEGs (i.e., with an absolute value of log_2_(fold change) > 2) comparing treatment effects was generated using normalizing reads. Furthermore, gene ontology (GO) enrichment analysis of DEGs was conducted using the GOstats package in R, and the Kyoto Encyclopedia of Genes and Genomes (KEGG) pathway gene set enrichment analysis of DEGs was performed using the clusterProfile software.

Data processing for ribo-seq was similar to that for RNA-seq. In addition to removing low-quality reads and reads with > 10% N, rRNA and tRNA reads were filtered out by mapping all reads to the *A. thaliana* ribosomal RNA gene database using bowtie [35] with default parameters. Next, TopHat2 was used to align ribo-seq data to the *A. thaliana* reference genome, and unmapped multiexon spanning reads were subjected to spliced alignment [36]. Quantification of mapped results to gene level was performed using HTSeq with its default mode “union” [37]. The read counts were used to calculate the expected number of reads per kilobase of transcript sequence per million base pairs sequenced (RPKM) using FeatureCounts v1.5.0-p3. Normalized read counts were subjected to DEG analysis using the DESeq2 package in R. Genes with |log2 fold change (FC)|> 2 and adjusted *p*-value < 0.05 (Benjamini–Hochberg method) were considered significantly differentially expressed. These thresholds were selected to ensure both statistical rigor and biological relevance, as commonly applied in plant stress transcriptomics. [38]. For ribosome profiling (ribo-seq), reads were processed similarly to RNA-seq. Next, the translation efficiency (TE) of each gene (i.e., ribosome-protected mRNA fragments/mRNA) was calculated, and differentially transcribed genes (DTGs) as well as differential TE genes (DTEGs) between treatments were identified using both processed RNA-seq and ribo-seq data in R using the method described by [39], with |log2FC|> 2 and adjusted *p* < 0.05 as cutoffs. GO and KEGG enrichment analyses were conducted using the same methods used for the RNA-seq data.

#### Proteomics, metabolomics, and phytohormone analyses

Tandem mass spectra were extracted using the Proteome Discover software v2.4.1.15 (Thermo Fisher Scientific). The SEQUEST software was used for searching all MS/MS samples against the UniProt database (Taxonomy: *A. thaliana*, 121,719 entries) assuming trypsin as the digestion enzyme [40]. The searching parameters were set with a fragment ion mass tolerance of 0.020 Da and a parent ion tolerance of 10.0 PPM. Carbamidomethyl of cysteine and TMT pro plex of lysine and the N-terminus were specified in SEQUEST as fixed modifications. Oxidation of methionine was specified in Mascot as a variable modification [41]. Next, the percolator algorithm was used to control the peptide-level FDR to be < 1%. Peptides with at least one unique peptide were used for protein quantification, and the number of total peptides was used to normalize experimental bias. PCA was used to visualize the overall differences in expressed proteins between treatments and samples. Generalized linear models (GLMs) using the quasi-Poisson likelihood method were used to identify differentially expressed proteins between treatments, and an FDR method was used to correct *P*-values when computing from the quasi-Poisson model. Significantly expressed proteins were determined using an absolute value of log_2_(fold change) > 1 and an adjusted *P* value of ≤ 0.05. This approach accounts for technical variability and is suitable for label-free or TMT-based proteomics. We also conducted GO enrichment analysis of differentially expressed proteins.

The raw spectra data were converted into the mzXML format using ProteoWizard and processed with an R script for peak detection, extraction, alignment, and integration [42]. Next, the metabolites were annotated according to an MS2 database developed by BiotreeDB (v2.1) [43]. The cutoff for metabolite annotation was set at 0.3. Significantly altered metabolites and hormones were identified using GLM with FDR < 0.05. These metabolites were subjected to KEGG and PubChem databases for further confirmation. After obtaining the matching information, metabolic pathway analysis was conducted based on the pathway database of *A. thaliana* to identify metabolites associated with key cellular signaling and metabolic networks [44]. A network analysis was also performed using the differently expressed metabolites to explore the metabolic networks that respond to salt stimuli [45]. The phytohormone data were processed using the same workflow as that used for the metabolomics data. All statistical analyses (α = 0.05) and data visualizations were conducted in R (https://www.r-project.org) using the packages “factoextra,” “ggplot2,” “pacman,” “MetNet,” “MSnbase,” “CAMERA,” “FELLA,” and “ComplexHeatmap.”

## Results

We established a multiomics framework (Fig. 1) to explore the responses of *A. thaliana* seedlings to salt stress at the levels of transcripts, proteins, phytohormones, and metabolites, compared with untreated control plants. Transcriptome profiling was conducted 6 h after NaCl treatment using NGS to evaluate changes in gene expression. To analyze post-transcriptional regulation, ribosome profiling (ribo-seq) was performed to examine TE and start sites of. After 24 h of salt treatment, proteomic changes were evaluated using low-pH nano-HPLC–MS/MS to quantify total protein abundance. Concurrently, six key phytohormones, including auxin, ABA, GA_3_, cytokinin, SA, and JA, were quantified using HPLC–MS/MS, because these hormones play vital roles in plant growth and stress resistance. At 48 h after salt treatment, nontargeted metabolomics profiling was performed using UHPLC–QE–MS to obtain secondary metabolites in mock- and salt-treated *A. thaliana* seedlings.

### Transcriptomics analysis revealed a phase transition from normal growth to stress resistance in *A. thaliana* in response to salt treatment

**Fig. 2:**
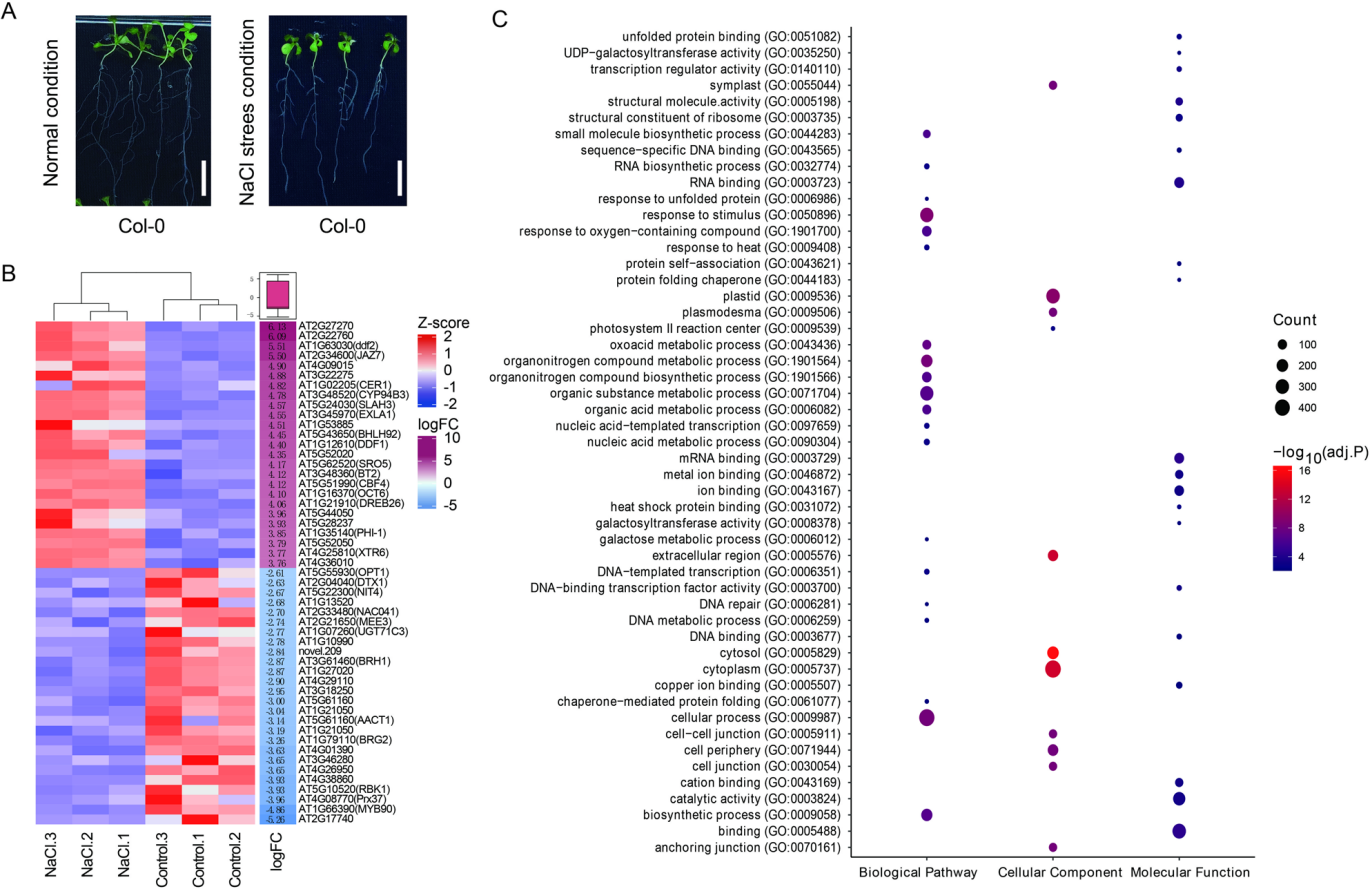
Plant phenotypes and transcriptional changes in early salt stress response. **A** Phenotypic comparison of *A. thaliana* Col-0 seedlings grown under control (1/2 MS medium) and salt stress (1/2 MS medium supplemented with 125 mM NaCl) conditions for 2 days. **B** Heatmap showing the top differentially expressed genes (DEGs) identified from RNA-seq analysis at 6 h post salt treatment. Genes with a log_2_ fold change (logFC) > 2 are displayed. Gene names and corresponding *A. thaliana* gene IDs are shown on the right. **C** Gene Ontology (GO) enrichment analysis of DEGs between salt-treated and control groups. The biological pathway, cellular component, and molecular function categories are shown in the figure. The size of dots represents the number of genes per GO term, and the color of dots represents the statistical significance as indicated by -log10(adjusted *P*-values)

*A. thaliana* is a salt-sensitive plant that often exhibits growth inhibition under salt stress. In our study, 2-week-old seedlings exposed to 125 mM NaCl for 6 h exhibited characteristic salt stress phenotypes, including smaller leaves, reduced root length, and fewer root hairs, compared with seedlings grown under control conditions (Fig. 2A). To identify the molecular mechanisms of *A. thaliana* in response to salt, we conducted transcriptomics analysis of salt- and mock-treated seedlings. After de novo assembling, 33,061 transcripts corresponding to 23,132 annotated gene loci in *A. thaliana* were identified (Supplemental Table 1). PCA revealed a clear separation between salt-treated and control biological replicates (Supplemental Fig. 1A), indicating different transcriptional responses to salt stress. Of the 33,061 transcripts, 3087 were differentially expressed at 6 h post treatment, including 1469 downregulated and 1618 upregulated transcripts (Supplemental Table 2). The top 50 DEGs, including 25 most upregulated and 25 most downregulated, were visualized in a heatmap (Fig. 2B). Among the important upregulated genes, the JA signaling repressor *JAZ7* (At2G34600) exhibited a five-fold increase in expression under salt treatment, which is consistent with previous findings [46]. The expression level of C-repeat-binding factor 4 (CBF4), a member of the dehydration-responsive element-binding family, increased by more than four-fold after salt treatment (Fig. 2B), suggesting its role in attenuating ABA and salt responses in *A. thaliana* [47]. The basic helix–loop–helix (bHLH) transcription factor *bHLH92* (At5G43650) was upregulated by approximately 4.5-fold, similar to that reported in a previous study [48]. Conversely, salt treatment resulted in a significant downregulation of a NAM, ATAF1/2, CUC2 (NAC) transcription factor *NAC041* (At2G33480) and *MYB90/PAP2*, whose levels were reduced by 2.7- and 4.86-fold, respectively. These genes are implicated in stress signaling and anthocyanin synthesis [49–51], emphasizing the impact of salt stress on both defense pathways and secondary metabolism.

GO enrichment analysis of DEGs between mock- and salt-treated *A. thaliana* samples (Fig. 2C) revealed significant enrichment in several biological processes related to transcription, DNA templates (GO:0006351), stress responses to water deprivation (GO:0009415), wounding (GO:0009611), general stress (GO:0006950), and response to oxygen-containing compounds (GO:1,901,700). Additional enriched terms were associated with responses to chemical stimuli, such as lipids (GO:0033993), hormones (GO:0009725), endogenous stimuli (GO:0009719), and general chemical stimuli (GO:0042221) (Fig. 2C). At the molecular function level, DEGs were significantly associated with double-stranded DNA binding (GO:0003690), DNA-binding transcription factor activity (GO:00037000), and general DNA binding (GO:0003677). For cellular component categories, DEGs were primarily localized to the nucleus (GO:0005634) and membrane (GO:0016020). These data suggest strong transcriptional changes and stress responses in salt-treated plants. To further investigate the interaction networks, we subjected all DEGs and the top 50 DEGs to STRING network analysis (Supplemental Figs. 2 and 3; Supplemental Table 3). The key genes included *JAZs*, *LOXs*, *OPR3*, *RAS1*, and *P5CS*, with *OPR3* and *P5CS1* acting as critical components of JA and proline biosynthesis pathways, both of which are essential for adaptation to salt stress [52, 53]. The KEGG pathway analysis further identified the enrichment of DEGs in the plant hormone signal transduction and plant–pathogen interaction pathways (Supplemental Figs. 4 and 5). In particular, PP2Cs and ABFs from the ABA pathway were significantly upregulated under salt stress, consistent with previous reports (Supplemental Fig. 4). Conversely, several plant–pathogen interaction genes, including *FLS2*, *FER*, and *MPK4*, were downregulated, suggesting a trade-off between abiotic (salt) and biotic stress responses.

### Ribo-seq analysis revealed diverse post-transcriptional regulatory responses to salt stress in *A. thaliana*

**Fig. 3:**
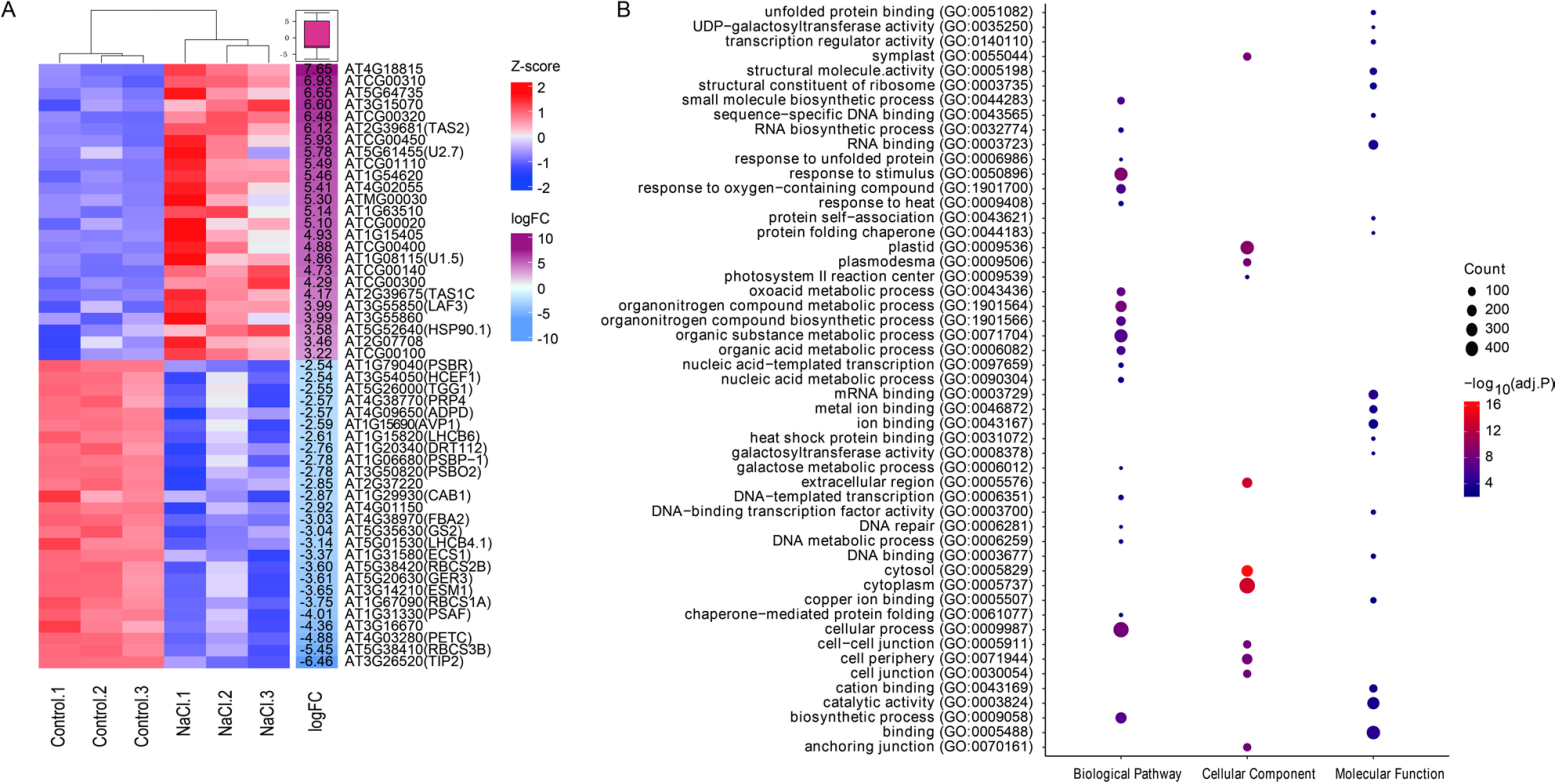
Ribo-seq analysis reveals translational changes in response to salt stress. **A** Heatmap showing differentially translated genes in *A. thaliana* following 12-h salt treatment. Genes with a log2FC > 2 are displayed. Gene names and corresponding *A. thaliana* gene IDs are shown on the right. **B** GO enrichment analysis of differentially translated genes between mock- and salt-treated samples. Enriched terms include biological pathway, cellular component and molecular function are shown in the figure. Dot size represents the number of genes per GO term, and color indicates statistical significance as − log_10_ (adjusted *P*-values)

To capture early translational regulation beyond transcriptional shifts, we performed ribosome profiling at 12 h after salt treatment in *A. thaliana* [54]. PCA revealed a distinct separation between treatments, with tighter clustering among salt-treated replicates (Supplemental Fig. 1B). Ribo-seq provided a more accurate representation of actively translated genes than RNA-seq (Supplemental Table 4). We identified 748 DTGs under salt stress conditions (Supplemental Table 5). A heatmap of the top translationally regulated genes (baseMean > 50 and |log_2_FC|> 2) is depicted in Fig. 3A. Remarkably, transacting small interfering RNAs, *TAS2* (At2G39681) and *TAS1C* (At2G39675), along with small nuclear RNAs, *U2.7* (At5G61455) and *U1.5* (At2G39675), exhibited increased translational activity under salt stress conditions, which is consistent with previous findings [55, 56]. Furthermore, the chaperone *HSP90.1* (At5G52640) was upregulated, potentially influencing polyamine metabolism and reactive oxygen species homeostasis under salt stress [57]. However, some genes known to positively regulate salt stress demonstrated decreased TE. For instance, aquaporin *TIP2* (At3G26520), which contributes to the transcriptional regulation of stress response genes, showed significantly reduced translation (log_2_FC = − 6.46), despite its known role in salt tolerance [58]. Moreover, multiple genes associated with photosystem and chloroplast functions, including *PsbR*, *PsbP, CFBP1*, *HCEF1*, *PsbO*, *CAB1*, and *ATPD*, failed to undergo translation (Fig. 3A), suggesting a shift from plant vegetative growth to a defensive stage by suppressing chloroplast development under salt stress. GO enrichment of the actively translated genes (Fig. 3B) emphasized biological processes such as responses to stimulus (GO:0050896), organic substance metabolic processes (GO:0043436; GO:1,901,564; GO:0071704), cellular processes (GO:0009987), and biosynthetic processes (GO:0009058) in response to salt treatment. The enriched cellular components primarily involved the cytoplasm, plastids, cytosol, and extracellular regions, whereas molecular functions were dominated by binding activity (RNA and ion binding) and catalytic activity. These findings demonstrate a post-transcriptional regulatory mechanism under salt stress, characterized by the suppression of growth-related pathways and selective enhancement of defense-related translation in *A. thaliana*.

### Proteomics analysis demonstrated differential protein accumulation in *A. thaliana* in response to salt stress

**Fig. 4:**
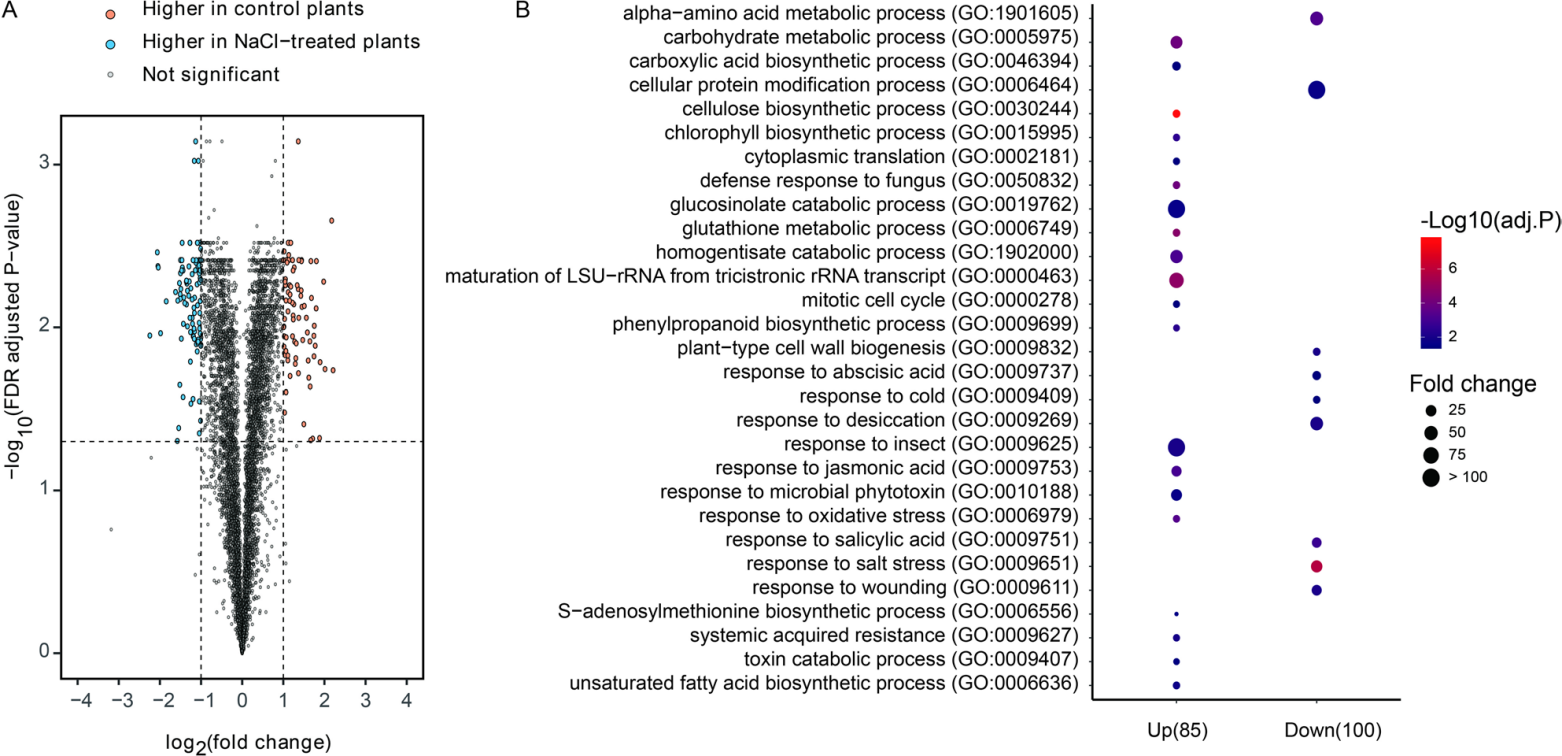
Proteomic analysis reveals protein abundance changes in response to salt stress. **A** Volcano plot of differentially expressed proteins between mock- and salt-treated *A. thaliana* samples at 24 h post-treatment. Blue dots represent downregulated proteins, and yellow dots represent upregulated proteins under salt stress. Proteins with a |log2FC|> 2 are displayed in the plot. **B** GO enrichment analysis of upregulated and downregulated proteins from proteomic analysis. Dot size corresponds to the number of proteins enriched in each GO term, and dot color indicates statistical significance based on -log_10_ (adjusted *P*-value)

With translational patterns defined, we proceeded to 24 h to examine the resultant proteome and to link protein abundance to the hormonal shift. PCA revealed a clear separation between mock- and salt-treated samples (Supplemental Fig. 1C). Label-free shotgun proteomics data identified 185 significantly altered proteins between treatment groups (Supplemental Table 6). As illustrated in the volcano plot (Fig. 4A), 85 proteins were significantly upregulated, whereas 100 proteins were downregulated after salt treatment. GO analysis revealed distinct protein clusters, with a remarkable downregulation in proteins associated with S-adenosylmethionine (AdoMet) biosynthesis (GO:0006556). This inhibition hindered AdoMet recycling, affecting the synthesis of ethylene, thermospermine, spermine, spermidine, and putrescine from the 5ʹ-methylthioadenosine (MTA) cycle. Furthermore, the unsaturated fatty acid biosynthetic process (GO:0006636) in *A. thaliana* was suppressed under salt stress, as detailed in Supplemental Table 7. Previous research has linked fatty acid regulation with plant hormones such as ABA, auxin, and JA, which impact defense signaling pathways, such as nitric oxide (NO) accumulation, hypersensitive response (HR), and ROS production reduction (Fig. 4B). Conversely, upregulated proteins under salt stress were primarily associated with catabolic and responsive processes (Supplemental Table 7), indicating a transition from plant growth development to a responsive stage to external stimuli or environmental changes. As indicated by the above-described data, the abundance of early response elements, including DNA-binding proteins, double-stranded DNA-binding proteins, and transcriptional activity proteins, was diminished. Hence, downstream proteins, such as those involved in the biosynthesis of unsaturated fatty acids (GO:0006636), response to microbial phytotoxin (GO:0010188), and response to desiccation (GO:0009269), were upregulated. Consistent with previous findings, the metabolic processes suggest a future accumulation of metabolites, indicating potential significant differences. Pathways associated with carbohydrate metabolic processes (GO:0005975), carboxylic acid biosynthetic process (GO:0046394), and alpha-amino acid metabolic process (GO:1,901,605) were significantly upregulated under salt stress, providing strong evidence that an increased accumulation of primary and secondary metabolites occurs in response to salt stress in *A. thaliana*.

### Transcriptomics, ribosome profiling, and proteomics analyses revealed multiple reactions of certain types of genes associated with salt stress

**Fig. 5:**
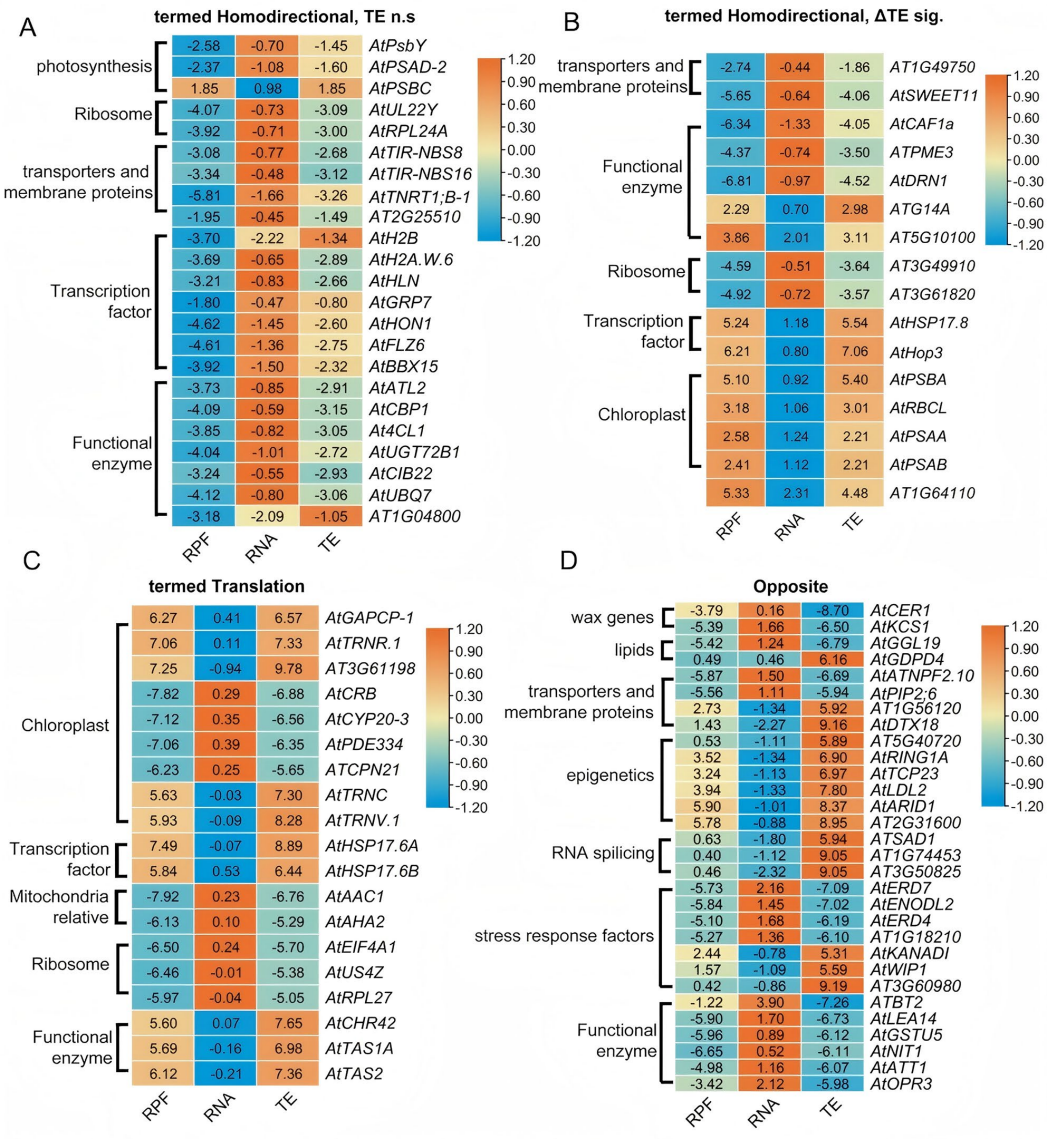
Combined analysis of RNA-seq, ribo-seq, and proteomeo-seq indicated 48-h dynamic changes in salt response in *A. thaliana*. Line charts reflecting the classical patterns of different groups in Supplemental Fig. 6A. Three different stages of FKPM of certain groups of DEGs are shown for comparison. **A** GO enrichment analysis of the differentially expressed genes of temporal dependent RNA-seq, ribo-seq, and proteomics sample groups from termed “Homodirectional TE n.s”. This diagram showed the genes and proteins which showed similar trends but in RPF and TE group, they did not show significant difference. **B** GO enrichment analysis of the differentially expressed genes of temporal dependent RNA-seq, ribo-seq, and proteomics sample groups from termed “Homodirectional TE sig.”. This diagram showed the genes and proteins which showed similar trends but in RPF and TE group, and they showed significant difference in all three omics. **C** GO enrichment analysis of the differentially expressed genes of temporal dependent RNA-seq, ribo-seq, and proteomics sample groups from “Translation.” The group of samples are genes response to salt at the translation level rather than at the transcriptional and post-transcriptional levels. The upregulated and downregulated proteins are shown in the figure. The size of the dots indicates the count numbers, and the colors of the dots indicate the − log10-adjusted *P*-values. **D** GO enrichment analysis of the differentially expressed genes of temporal dependent RNA-seq, ribo-seq, and proteomics sample groups from “Opposite.” The group of samples are genes response to salt but at translation level are different patterns than that in RNA-seq and ribo-seq data. The upregulated and downregulated proteins are shown in the figure. The size of the dots indicates the count numbers, and the colors of the dots indicate the -log_10_ (adjusted *P*-values)

In general, the regulation of transcription, post-transcription, and translation in plants in response to specific stimuli follows a temporal–spatial pattern. In our integrative analysis of RNA-seq, ribo-seq, and proteomic data, some DEGs exhibited consistent changes under salt stress, whereas others did not. To better understand these patterns, we categorized all DEGs into the following four groups: unchanged, homodirection with no statistically significant changes, homodirection with significant changes, and oppositely significantly changed (Supplemental Tables 8–12). Remarkably, 1821 genes exhibited different degrees of expression in transcription, post-transcription, and translation (Supplemental Table 8). A total of 23 genes exhibited synchronized changes, but these were not statistically significant (Supplemental Table 9) ($\Delta$ TE n.s, termed homodirectional but no significant difference). These genes can be categorized into photosynthesis, ribosome, membrane proteins, transporters, transcription factors, and functional enzymes (Fig. 5A). Several transporters, such as AtNRT1;B-1, TIR-NBS8, and TIR-NBS16, consistently demonstrated decreased expression across transcriptional, post-transcriptional, and protein levels. Genes and proteins of histones, such as AtH2B and AtH2A.W.6, also exhibited similar trends of reduced expression.

Another 16 genes exhibited some differences (ΔTE sig., termed homodirectional and statistically significant changes; Fig. 5B; Supplemental Table 10). Interestingly, several chloroplast-related genes and proteins demonstrated trends of increased expression across all three levels after salt stress. In both $\triangle$ TE n.s and ΔTE sig. groups (Figs. 5A and 5B), genes and proteins related to ribosome-related genes showed reduced expressions at transcriptional and protein levels.

In the “Translation” group (433 DEGs; Supplemental Table 11), significant changes appeared only in the ribo-seq and proteomics data, not in RNA-seq data (Supplemental Table 11) (termed Translation). We selected the top differentiated genes and proteins to generate a heatmap for this group (Fig. 5C). The heatmap revealed significant upregulation of chloroplast tRNAs. Specially AtRNR.1, AtTRNC, and ArTRNV.1 showed significant upregulation in response to salt stress at post-transcriptional and translational levels, consistent with major gene and protein response in the chloroplast. Almost all ribosomal proteins showed decreased expression trends and were consistently downregulated (Fig. 5). Two heat shock protein (HHSP) chaperones showed no difference in RNA-seq data at the transcript level; but their expression was significantly upregulated in ribo-seq proteomic analyses.

In the “Opposite” group (246 DEGs; Supplemental Table 12), transcript and protein levels displayed opposite trends in RNA-seq, ribo-seq, and proteomics analyses. In the “∆TE n.s” group, the 23 genes included those related to photosynthesis, ribosome, membrane proteins, transporters, transcription factors, and functional enzymes (Fig. 5A). Many transporters, such as AtNRT1;B-1, TIR-NBS8, and TIR-NBS16, showed reduced expression at transcriptional, post-transcriptional, and protein levels. Histone genes and proteins, such as AtH2B and AtH2A.W.6, also demonstrated similar downregulation. Another 16 genes could be grouped similarly “∆TE sig.” (Fig. 5B). Genes and proteins related to chloroplast activity demonstrated increased expression trends in all analyses after salt stress. In both groups shown in Figs. 5A and 5B, ribosome-related genes and proteins showed reduced at post-transcriptional and protein expression. We also identified genes and proteins, like AtCER1, AtNIT1, AtATT1, and AtKCS1, which were involved in wax biosynthesis and metabolic enzymatic function. These were upregulated transcriptionally during early salt stress but were strongly repressed at translational and protein levels during prolonged salt stress. Several genes and proteins related to epigenetic regulation and RNA splicing also showed reversed expression trends.

A scatter plot showed the association between DEGs and the three datasets (Supplemental Fig. 6A). GO enrichment analysis of the 433 DEGs in the “Translation” group indicated a significant enrichment in catalytic activity (GO:0003824) under molecular function, which suggests increased protein synthesis and metabolism due to salt stress (Supplemental Fig. 6B). The biological pathway was mainly related to cellular metabolic process (GO:0044237), metabolic process (GO:0008152), and organic substance metabolic process (GO:0071704). For cellular component, DEGs were mostly associated with cellular anatomical entity (GO:0110165), cytoplasm (GO:0005737), and plastid (GO:0009536). In contrast, GO analysis of the 246 DEGs in the “Opposite” group did not find significant enrichment of molecular function terms. This finding suggests a nonlinear regulatory link among transcription, post-transcription, and protein levels under salt stress (Supplemental Fig. 6C). However, in the biological pathway, DEGs were mainly enriched in response to stress (GO:0006950), response to chemicals (GO:0042221), and response to oxygen-containing compounds (GO:1,901,700). The cellular component terms of this opposite group included endomembrane system (GO:0012505), plasma membrane (GO:0005886), and vacuole (GO:0005773). These findings emphasize the complex and dynamic regulatory network in plants, where transcriptional, post-transcriptional, and translational responses to abiotic stress are not always consistent in adaptation to salt stress.

### Stress response-related phytohormones responded differently to salt treatment in A. thaliana

**Fig. 6:**
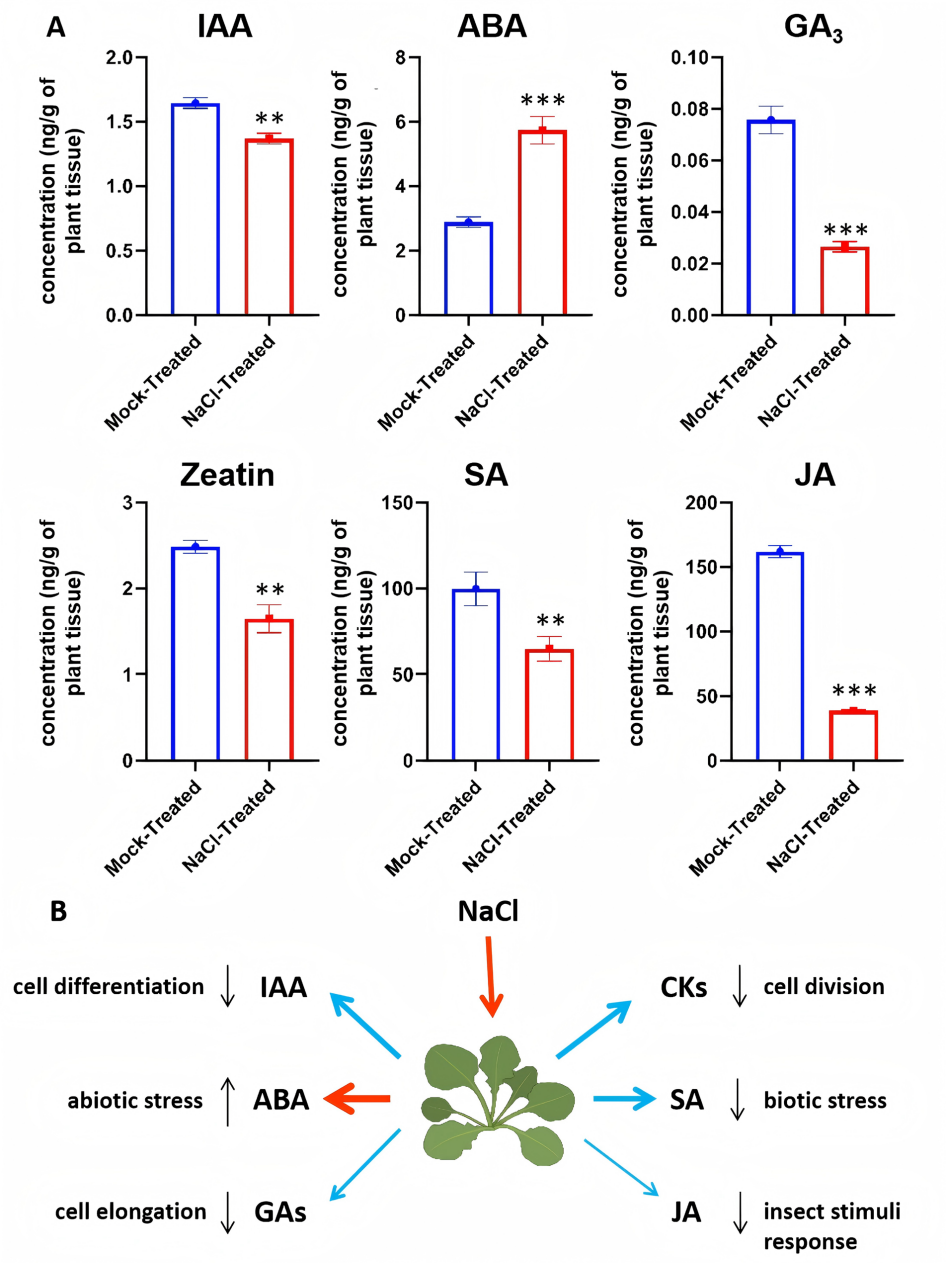
Plant hormones in response to salt stress in *A. thaliana*. **A** Quantitative analysis of six major phytohormones, including indole-3-acetic acid (IAA), abscisic acid (ABA), gibberellic acid (GA3), zeatin (cytokinin), salicylic acid (SA), and jasmonic acid (JA), in mock- and salt-treated *A. thaliana* seedlings. Hormone levels were measured 24 h post-treatment. **B** Schematic representation of phytohormone alterations under salt stress. Salt exposure led to an increase in ABA levels, while IAA, GA3, zeatin, SA, and JA were downregulated, reflecting a shift from growth-promoting to stress-responsive signaling

Concurrently at 24 h, we quantified six phytohormones to test whether the proteome changes coincide with phytohormones’ alteration. Accumulating evidence suggests that phytohormones are key regulators in facilitating the adaptation of plants to adverse conditions and also regulating the growth and development of plants [6]. In our study, we observed significant alterations in the plant hormone signal transduction pathway under salt stress through transcriptional analysis (Fig. 2C and Supplemental Fig. 3, Supplemental Table 3). To further characterize plant phytohormone profiles in *A. thaliana* under salt stress in the same time frame of collecting the proteomics data (i.e., 24 h after salt treatment), we quantified the levels of six major phytohormones that are associated with plant growth and biotic and abiotic stresses (Fig. 6 and Supplemental Table 13). As shown in Fig. 6, the levels of the growth-regulating hormone IAA significantly reduced after salt treatment compared with those in plant samples in the control group. Similarly, growth-promoting plant hormones such as cytokinin (zeatin) and GA_3_ exhibited decreased levels in salt-treated samples, indicating a transition from growth to stress defense in *A. thaliana*. In contrast, the salt- and drought-related plant hormone ABA showed two-fold increased levels compared with those in mock-treated samples, consistent with previous reports [6]. The other two stress-responsive hormones, JA and SA, were downregulated in salt-treated seedlings compared with those in control seedlings. Remarkably, salt stress significantly suppressed JA levels to one-third of that in the control, emphasizing the dose-dependent and flexible nature of SA and JA under salt stress. This evidence also suggests that the JA biosynthetic process is repressed under salt stress. For instance, *AtOPR3* (At2G06050), which catalyzes the transfer of OPDA into JA [59], initially demonstrated transcriptional induction in early salt response. However, its protein levels declined by six-fold under salt exposure, correlating with the observed reduction in JA signals (Supplemental Table 8). Similarly, the expression of RGA1 (AT2G01570), which encodes a DELLA protein in response to GA signals, increased at the transcriptional level but decreased at the protein level, causing the reduction of GA signals under salt stress conditions (Supplemental Table 8).

### Prolonged salt stress resulted in the accumulation of multiple metabolites that contribute to increased salt tolerance in A. thaliana

**Fig. 7:**
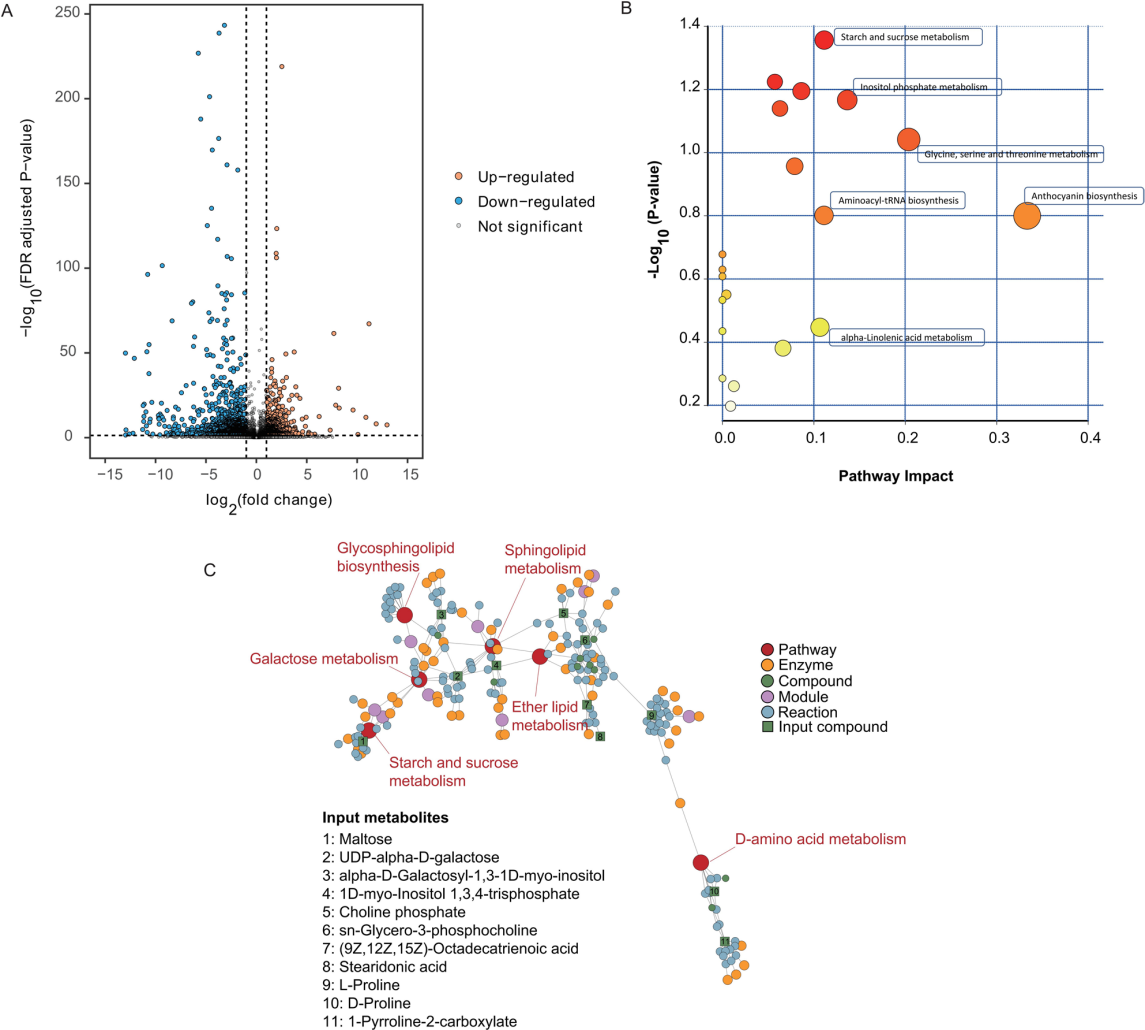
Metabolomic analysis revealed major plant metabolite alterations in response to salt stress in *A. thaliana*. **A** Volcano plot showing differentially regulated metabolites between mock- and salt-treated samples. Blue dots represent significantly downregulated metabolites, and yellow dots represent significantly upregulated metabolites under salt stress. The x-axis indicates log_2_ fold change, and the y-axis shows -log_10_ of the FDR-adjusted *P*-value. **B** Pathway enrichment analysis of significantly altered metabolites highlights major metabolic pathways affected by salt stress. **C** Network diagram of representative annotated metabolites altered under salt stress

Finally, we profiled the 48 h metabolome to determine how early transcriptional/post-translational events ultimately translate into biochemical defense compounds that enhance long-term salt tolerance, a critical period for metabolic and phenotypic alterations in response to salt stress (Fig. 2A). PCA revealed a clear separation between the metabolite profiles of mock- and salt-treated samples, indicating distinct metabolic states (Supplemental Fig. 1D). In total, 2519 metabolites exhibited significant changes after salt treatment compared with controls (Supplemental Table 14), among which 1780 metabolites showed decreased expression by at least two-fold, whereas 739 metabolites showed increased expression by at least two-fold (Fig. 7A; Supplemental Table 14). These metabolites were categorized into several major pathways, including starch and sucrose metabolism, inositol phosphate metabolism, glycine/serine/threonine metabolism, aminoacyl-tRNA biosynthesis, anthocyanin biosynthesis, and alpha-linolenic acid metabolism (Fig. 7B). From the top 53 annotated metabolites, we constructed a network illustrating the metabolic reprogramming by salt stress (Fig. 7C and Supplemental Table 15). Remarkably, d-proline and 1-pyrroline-2-carboxylate, which are involved in plant stress defense responses, were significantly enriched in salt-treated plants. Furthermore, maltose, which probably contributes to osmotic pressure and ROS-related responses, was activated in salt-stressed samples. Altogether, these findings provide evidence of a dynamic shift in the metabolome that supports the adaptation of *A. thaliana* to salt stress.

## Discussion

Salt stress poses a significant threat to plant biomass and crop yield [5]. Elucidating the molecular mechanisms that underlie plant responses to salt stress during the first 48 h is essential for understanding plant growth regulation and abiotic-stress adaptation. Previous studies have established key phases of the *A. thaliana* salt-stress response: an early transcriptional burst within 6 h [60], translational and hormonal reprogramming peaking at 12 h [61], a proteome switch from quiescence to recovery at 24 h [62], and late accumulation of osmolytes and secondary metabolites by 48 h [63]. In this study, we used a multiomics approach to dissect the dynamic changes and temporal responses of *A. thaliana* to salt stress focusing on four early time-points (6, 12, 24, and 48 h). Our results demonstrated that during the initial 6 h of salt treatment, transcriptional responses primarily occurred in the nucleus and membrane, suggesting early Na^+^ signal transduction and modulation of DNA-binding transcription factors involved in regulating plant growth and defense pathways (Fig. 2). By 12 h, these transcriptional activities transitioned to post-transcriptional regulation, as evidenced by differences in TE revealed through ribosome profiling (Fig. 3). At 24 h, the salt-induced signals influenced protein abundance and plant hormone levels, including the activation of second messenger protein kinase cascades (Figs. 4 and 6). A subsequent 24 h period is required for these upstream regulatory events to transduce into metabolomic changes, ultimately resulting in alterations to plant phenotypic traits (Fig. 7). Important metabolite changes included tRNAs, amino acids, starch, and sucrose, all of which are crucial elements for supporting plant growth. A integrative schmatic diagram was generated to show the key findings in our study (Supplemental Fig. 7). However, future studies should examine the intermediate- and long-term phases governing leaf expansion, osmotic adjustment, and final biomass.

The classical salt overly sensitive signaling pathway reveals how Na^+^ signals are transmitted from the membrane into the cell. In this process, SOS1, SOS2, and SOS3 work together to trigger subcellular reactions that expel Na^+^ into the cytoplasm [3]. Our data expand this model by revealing that salt stimuli also elicit rapid transcriptional responses within the nucleus (Fig. 2C). In particular, genes involved in JA signaling, such as *OPR3* and *JAZ7*, exhibited significant changes in expression (Supplemental Fig. 3). These genes and their coexpression patterns inhibited JA biosynthesis and signaling transduction. For instance, *LOX3* expression increased by 2.29-fold compared with control samples (Supplemental Table 2), supporting previous evidence that LOX3 positively regulates salt tolerance, as *lox3* mutants are hypersensitive to salt stress but are rescued by MeJA treatment [10]. The negative regulator of JA signaling *JAZ7* was upregulated by more than five-fold in salt-treated samples (Fig. 2C), further contributing to the reduction of JA signals [64]. OPR3, an enzyme that uses 4,5-didehydrojasmonate to generate JA in vivo, contributes to the inhibition of JA signal transduction [52]. These genes collectively modulate JA signaling, promoting the generation of JA-Ile and MeJA in response to salt stress. At 24 h after treatment, the plant transitioned to another phase of defense. The reduction of AdoMet observed in iTRAQ data confirmed decreased abundance of polyamines in salt-stressed samples. This decline probably impairs antioxidant enzyme function, polyamine metabolism, and oxidative stress tolerance [65]. By 48 h of salt treatment, the biosynthesis of sucrose, inositol, tRNAs, amino acids, and anthocyanin occurs, suggesting adjustments in cellular components and metabolites in response to salt stress. These adjustments support earlier findings linking anthocyanin accumulation with salt stress [66].

Salt responses in *A. thaliana* emerge from coordinated but sometimes antagonistic signaling among ABA, JA, SA, and ethylene. Consequently, increases in one pathway may down-modulate another, producing apparent inverse trends that are not strictly causal. We therefore interpret associations between pathway markers and phenotypes as consistent with—but not proof of—direct regulatory effects. Where possible, we examined coherence across layers (transcript → Ribo-seq → protein → metabolite) and timepoints to reduce the risk of attributing cross-talk to a single pathway. We also note that developmental stage and circadian phase can shift hormone baselines; our time-matched design reduces, but does not eliminate, these influences.

The multiomics approach allows us to identify key regulatory elements, including genes, proteins, and metabolites, across different biological processes and time points during stress exposure, which would be difficult to determine through a single omics analysis [67]. For instance, early transcriptional responses involving specific transcription factors may initiate a cascade of downstream effects that are later modulated at post-transcriptional and translational levels [68]. Changes in phytohormone levels further illustrate the crosstalk between signaling pathways and their effects on plant adaptation to salt stress [69]. However, integrating multiple omics presents several challenges. A major challenge is the complexity of data integration and interpretation [70], as each omics platform yields large and complex datasets with different types of variables and noise levels. When we integrated RNA-seq, ribo-seq, and proteomics data, we found different expression patterns at four time points of salt treatment in *A. thaliana* (Supplemental Fig. 6A). Another major challenge is the data QC and standardization, which are crucial for integration, demanding unified omics data standards and QC systems. For proteomics and metabolomics, we implemented stringent QC, MS/MS-verified identifications, blank filtering, and FDR correction to enhance robustness. Residual missing data and uncertainty may still remain. Furthermore, multiomics data analysis requires more robust computational and storage resources [71]. Differences in temporal and spatial resolution across omics platforms can hinder direct comparison across different levels of regulation [72]. For instance, a gene identified as differentially expressed at the transcriptional level may not necessarily exhibit corresponding changes at the protein level due to post-transcriptional regulation or technical limitations in protein detection (Supplemental Tables 8–12). Conversely, some proteins may exhibit significant changes in abundance without corresponding transcriptional alterations, emphasizing the importance of considering TE and post-translational modifications. Therefore, it would be necessary to optimize omics tools and validate findings through functional and phenotypical assays to advance plant science.

The integration of multiple omics approaches provides a comprehensive understanding of the trade-offs between normal growth and defense responses in plants under salt stress. Although this study delineates the dynamic changes in *A. thaliana* under salt stress across multiple tiers—from DNA to RNA, protein, hormone, and metabolic levels—the specific candidate genes and differential metabolites identified still require further experimental validation of their precise functions and underlying mechanisms. Modern molecular tools such as CRISPR-Cas and prime editing systems have advanced our ability to engineer desired traits at the DNA, RNA, or even protein level in plants [73]. Although research on salt stress has been prominent since the early 2000 s [3], relatively few studies have applied multiomics tools to explore salt tolerance mechanisms in *A. thaliana* [74]. Conversely, such approaches have been increasingly adopted in crops such as rice [75], legumes [76], wheat [77], and maize [78].

Although this study was performed in *A. thaliana*, the core regulatory nodes we identified have clear orthologues in major cereals. The key transcription factors we identified (*JAZ7*, *CBF4*, *bHLH92*) and the late-response metabolites (D-proline, 1-pyrroline-2-carboxylate) all have single-locus orthologs in rice, wheat, maize and soybean with > 65% average protein identity, which have been previously reported associated with salt tolerance in cereals [79, 80]. As a classical model plant, *A. thaliana* provides unique advantages for demonstrating foundational regulatory mechanisms that may be extrapolated to other plant species or crop systems. Beyond previously published studies on salt stress, our study deciphers the temporal dynamics of molecular responses in *A. thaliana* to salt exposure and establishes a working model for predicting abiotic stress responses in other plants.

## Conclusion

This study reveals a multilayered regulatory network in *A. thaliana*, in which salt stress triggers sequential transcriptional, post-transcriptional, proteomic, and metabolomic responses. The stage-specific hubs (*JAZ7*, *CBF4*, *bHLH92*) and late-stage metabolites (D-proline, 1-pyrroline-2-carboxylate) can serve as molecular markers for marker-assisted selection or as target loci for domestication in crops. Combining these markers with high-throughput phenotyping platforms will accelerate the breeding of salt-resilient cultivars.

## Supplementary Information

Supplementary Material 1.

## Data Availability

The RNA-seq data have been deposited in the National Center for Biotechnology Information (NCBI) under accession number PRJNA1305220. The ribo-seq data have been deposited in the National Center for Biotechnology Information (NCBI) under accession number PRJNA1354442.

## Abbreviations

ABA: Abscisic acid
NCED: 9-Cis-epoxycarotenoid dioxygenase
LOX3: Lipoxygenase 3
OPDA: 12-Oxo-phytodienoic acid
AP2/ERF: APETALA2/ethylene-responsive factor
JA: Jasmonic acid
NGS: Next-generation sequencing
ribo-seq: Ribosome profiling
CBF4: C-repeat-binding factor 4
bHLH: Basic helix–loop–helix
NAC: NAM, ATAF1/2, CUC2
AdoMet: S-adenosylmethionine
MTA: 5ʹ-Methylthioadenosine
NO: Nitric oxide
HR: Hypersensitive response
MS: Medium Murashige & Skoog medium
KEGG: Kyoto Encyclopedia of Genes and Genomes
DEG: Differentially expressed genes
PCA: Principal component analysis
GO: Gene ontology
RPKM: Reads per kilobase of transcript sequence per million base pairs sequenced
DTG: Differentially transcribed gene
